# Supplementary material for: Self-compliant ionic nanomesh for gas-permeable and stress-free on-skin electronics
Source: Nat Commun. 2025 Nov 20;16:11510. doi: 10.1038/s41467-025-66512-2 (PMC12749131; doi:10.1038/s41467-025-66512-2)
Supplement: Supplementary file 1 — Supplementary Information [file 41467_2025_66512_MOESM1_ESM.pdf]

Supplementary Information

# Self-compliant ionic nanomesh for gas-permeable and stress-free on-skin electronics

Qinqing Du (杜钦青)[1], Lingyan Liu (刘玲燕) [1], Shengtong Sun (孙胜童) [1]* & Peiyi Wu (武培怡)[1]*

[1]State Key Laboratory of Advanced Fiber Materials, College of Chemistry and Chemical Engineering & Center for Advanced Low-dimension Materials, Donghua University, Shanghai 201620, China.

Correspondence should be addressed to S.T.S. (email: shengtongsun@dhu.edu.cn) and P.Y.W. (email: wupeiyi@dhu.edu.cn)

## This file includes

**Suppl. Fig. 1.** Chemical synthesis of linear LCE

**Suppl. Fig. 2.** GPC curve of linear LCE

**Suppl. Fig. 3.** Schematic of benzophenone-induced chemical crosslinking in pLCE ionic nanomesh

**Suppl. Fig. 4.** Thickness of pLCE ionic nanomesh

**Suppl. Fig. 5.** SEM image of electrospun LCE ionogel

**Suppl. Fig. 6.** Temperature-sweep rheology of LCE films with/without ionic liquid

**Suppl. Fig. 7.** SEM image of ionic nanomesh produced at a sheath flow rate of 1.4 mL $h^{-1}$

**Suppl. Fig. 8.** SEM images and calculated porosities of pLCE nanomeshes at different core flow rates

**Suppl. Fig. 9.** SEM images of pLCE nanomeshes produced at different voltages

**Suppl. Fig. 10.** SEM images of pLCE nanomeshes produced at different collector rotation speeds

**Suppl. Fig. 11.** ATR-FTIR spectra of LCE core, PVP/AChCl sheath and pLCE ionic nanomesh

**Suppl. Fig. 12.** Heat-induced contraction of stretched pLCE ionic nanomesh

**Suppl. Fig. 13.** TGA curves of pLCE ionic nanomeshes fabricated with varying sheath flow rates

**Suppl. Fig. 14.** Oil permeability of pLCE ionic nanomesh

**Suppl. Fig. 15.** Low-field $^1$H NMR spectra of pLCE ionic nanomesh at different humidities

**Suppl. Fig. 16.** Tensile curves of LCE and PVP/AChCl films

**Suppl. Fig. 17.** Adhesion of pLCE ionic nanomesh to porcine skin

**Suppl. Fig. 18.** Tensile curve of pLCE ionic nanomesh

**Suppl. Fig. 19.** Notch resistance of pLCE ionic nanomesh

**Suppl. Fig. 20.** Loading-unloading and rheological curves of pTPU ionic nanomesh and TPU film

**Suppl. Fig. 21.** Scratch resistance of pLCE ionic nanomesh

**Suppl. Fig. 22.** Cyclic 90° peeling curves of pLCE ionic nanomesh at different peeling strengths

**Suppl. Fig. 23.** Probe-tack curve of pLCE ionic nanomesh on porcine skin

**Suppl. Fig. 24.** CLSM images of cell proliferation in pLCE ionic nanomesh

**Suppl. Fig. 25.** L929 cytotoxicity results of pLCE ionic nanomesh

**Suppl. Fig. 26.** Real-time EMG monitoring with commercial Ag/AgCl gel electrodes

**Suppl. Fig. 27.** SNRs of pLCE ionic nanomesh electrodes during various thumb movements

**Suppl. Fig. 28.** SNRs of pLCE ionic nanomesh electrodes by repeated grasping of different objects

**Suppl. Fig. 29.** Real-time EMG recorded with pLCE ionic nanomesh electrodes on sweaty skin

**Suppl. Fig. 30.** Real-time ECG recorded with pLCE ionic nanomesh electrodes

**Suppl. Table 1.** Comparison of moisture permeability among typical breathable on-skin materials

**Suppl. References S1-S10**

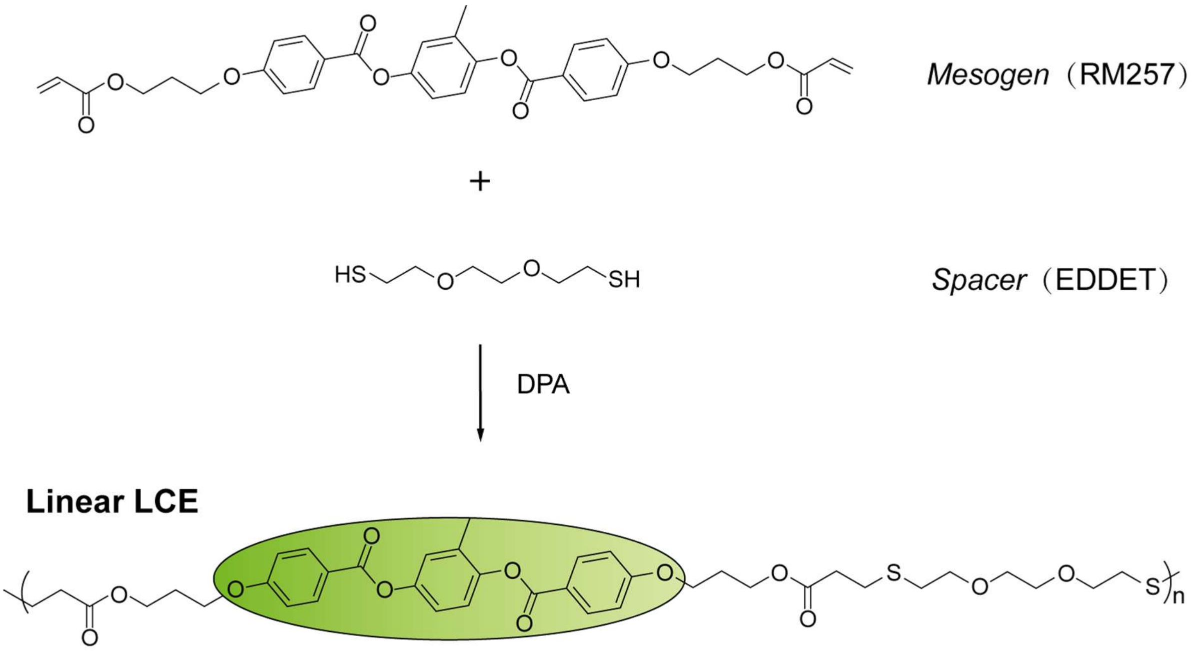


**Supplementary Fig. 1. Chemical synthesis of linear LCE.** Linear LCE was synthesized via a Michael addition reaction between the reactive mesogen, RM257, and the chain spacer, EDDET.

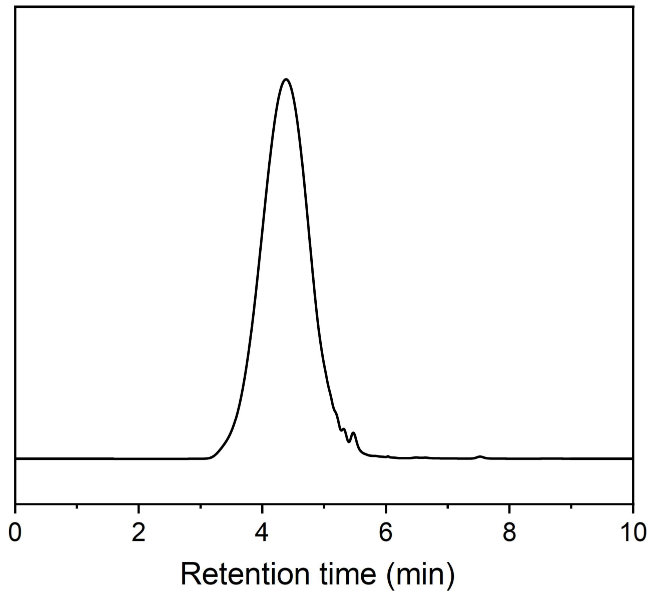


**Supplementary Fig. 2. GPC curve of linear LCE using THF as the eluent.** The sample for GPC was synthesized under the same conditions as the LCE spinning dope, but with TPU removed. The calculated number-average molecular weight is $3.4 \times 10^4$ g mol$^{-1}$, and the polydisperse index (PDI) is 2.3.

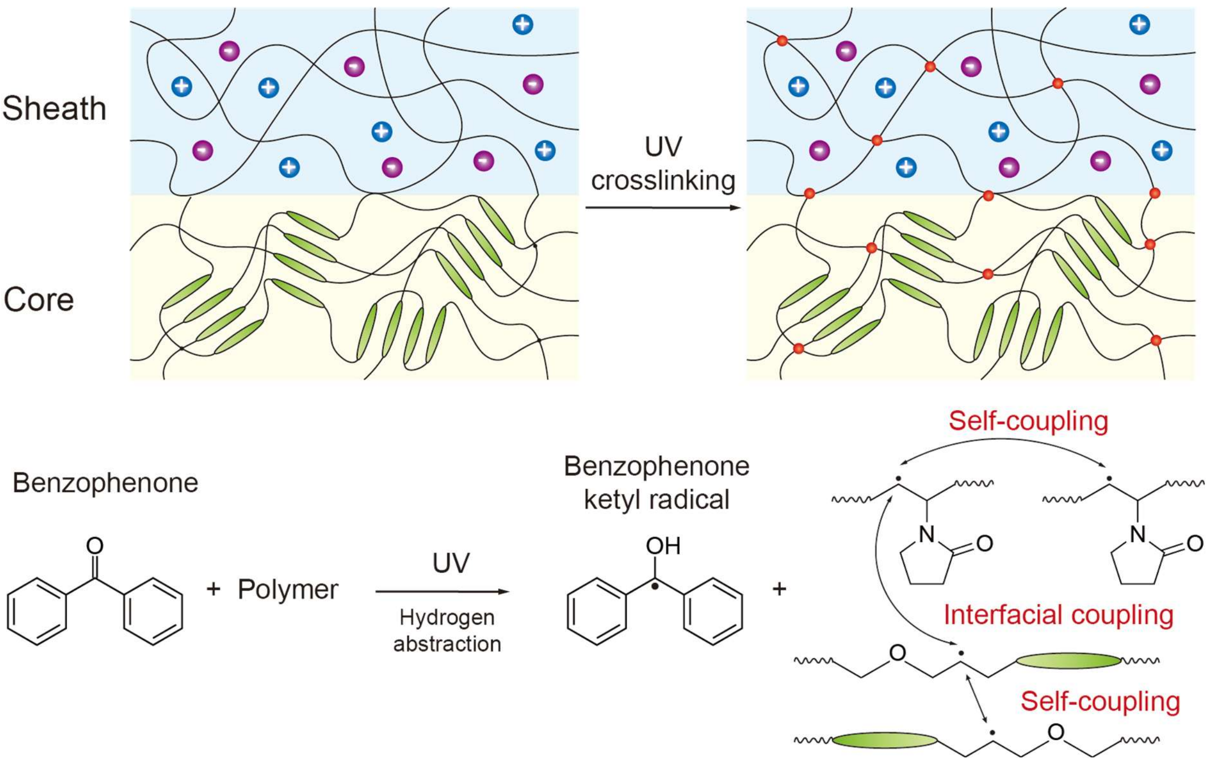


**Supplementary Fig. 3. Schematic of benzophenone-induced chemical crosslinking in pLCE ionic nanomesh.** Upon UV irradiation, excited benzophenone abstracts hydrogen atoms from PVP and LCE chains, forming benzophenone ketyl radicals. This process leads to both self-coupling (within PVP and LCE chains) and interfacial coupling (between core and sheath materials), resulting in chemically crosslinked PVP and LCE, and strong adhesion at the core-sheath interface.

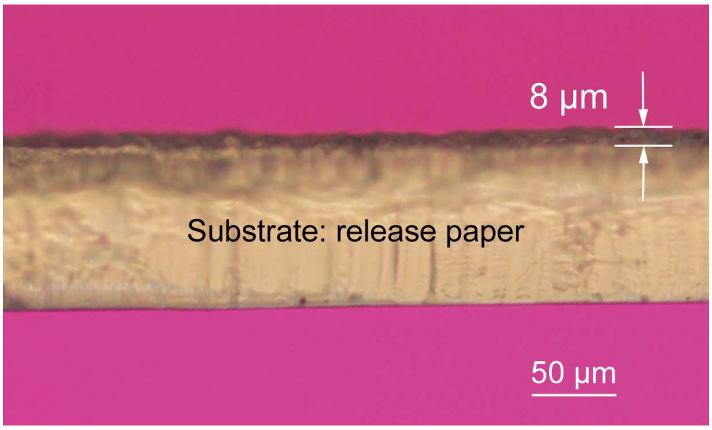


**Supplementary Fig. 4. Thickness of pLCE ionic nanomesh.** The thickness of the pLCE ionic nanomesh was measured to be ~8 μm, as determined by optical microscopy.

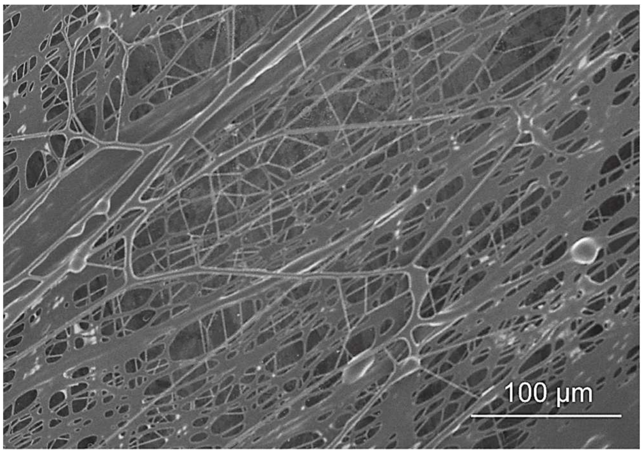


**Supplementary Fig. 5. SEM image of electrospun LCE ionogel.** Introduction of an LCE-compatible ionic liquid, 1-butyl-3-methylimidazolium hexafluorophosphate (BMIM $PF_6$, 50 mol% relative to the EDDET spacer), into the spinning dope allowed the fabrication of LCE ionogel nanomesh using the same procedure. However, the high polarity of the ionic liquid significantly deteriorated the quality of the resulting nanofibers.

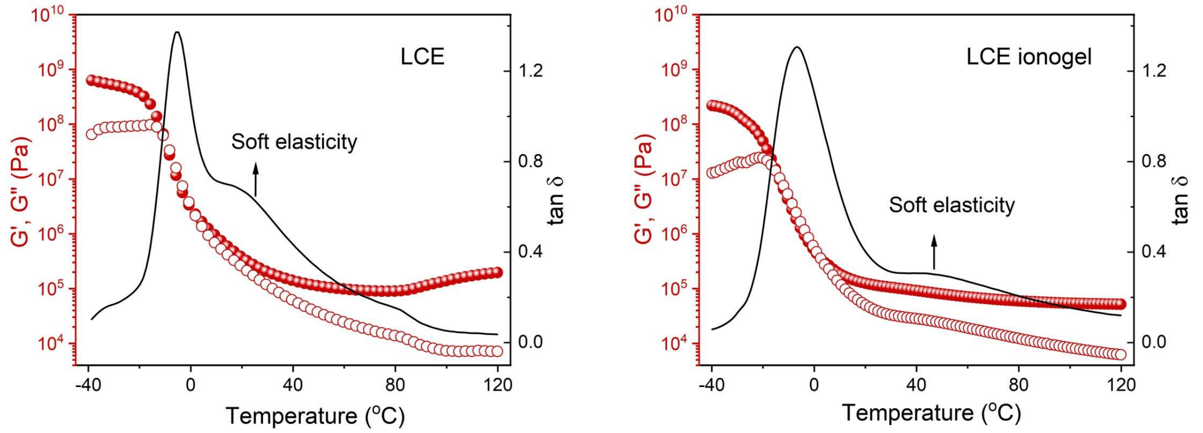


**Supplementary Fig. 6. Temperature-sweep rheology of LCE films with and without ionic liquid.** While the LCE film exhibited a clear tan δ peak around 25 °C, characteristic of soft elasticity, the introduction of BMIM $PF_6$ dramatically reduced this peak, indicating weakened soft elasticity. This is mainly attributed to the plasticizing effect of BMIM $PF_6$, which selectively binds to the soft spacers of the LCE and partially disrupts the collective assembly of mesogens[S1].

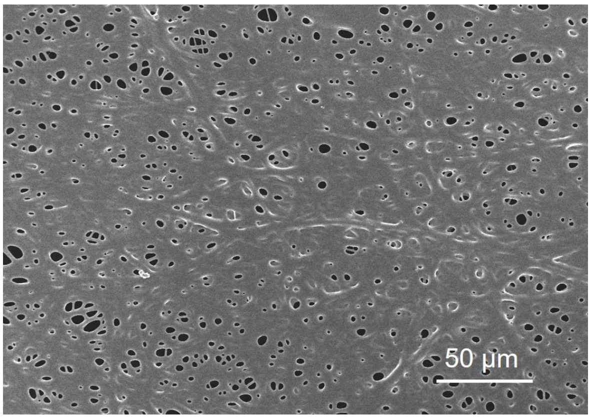


**Supplementary Fig. 7. SEM image of the ionic nanomesh produced at a sheath flow rate of 1.4 mL h$^{-1}$** (core flow rate = 0.3 mL h$^{-1}$). At this flow rate, the sheath layer was too thick, leading to an almost complete loss of nanomesh porosity.

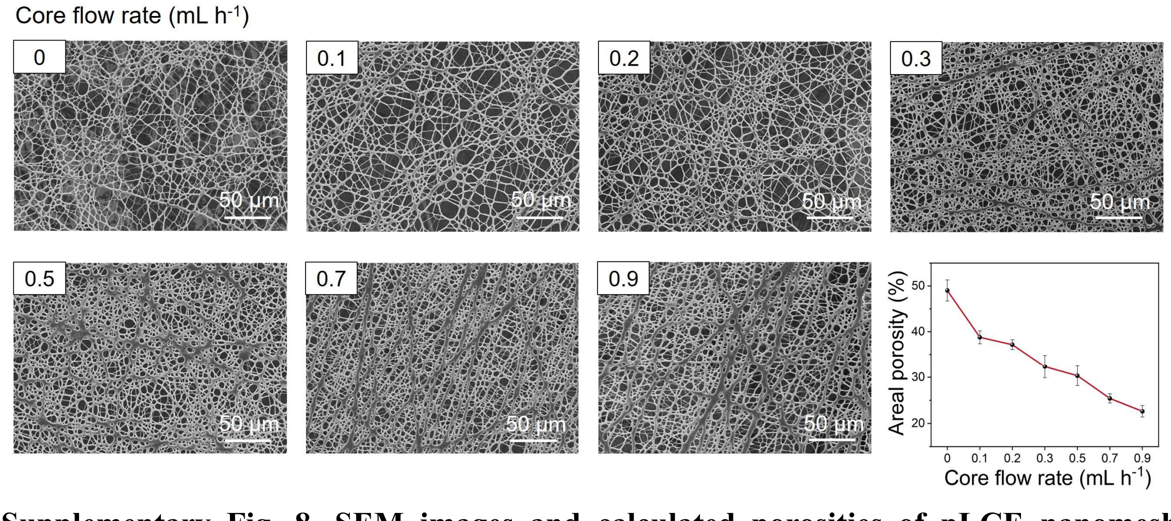


**Supplementary Fig. 8. SEM images and calculated porosities of pLCE nanomeshes produced at different core flow rates.** The sheath flow rate was fixed at 0.4 mL h$^{-1}$. Increasing the core flow rate from 0 to 0.9 mL h$^{-1}$ led to the growth of LCE core, resulting in a gradual reduction of areal porosity from 49% to 23%.

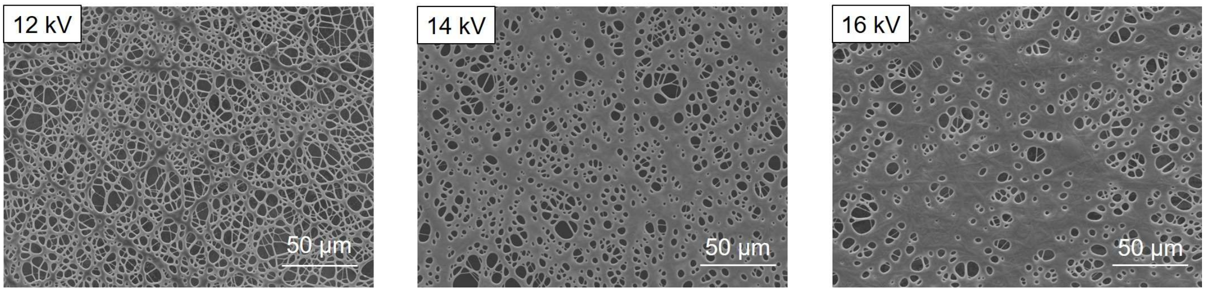


**Supplementary Fig. 9. SEM images of pLCE nanomeshes produced at different voltages** (core flow rate = 0.3 mL $h^{-1}$; sheath flow rate = 0.4 mL $h^{-1}$). A voltage of 12 kV was used in this study. Increasing the voltage to 14 and 16 kV led to excessively fast nanofiber deposition, leaving insufficient time for UV crosslinking, and the fibers tended to self-fuse.

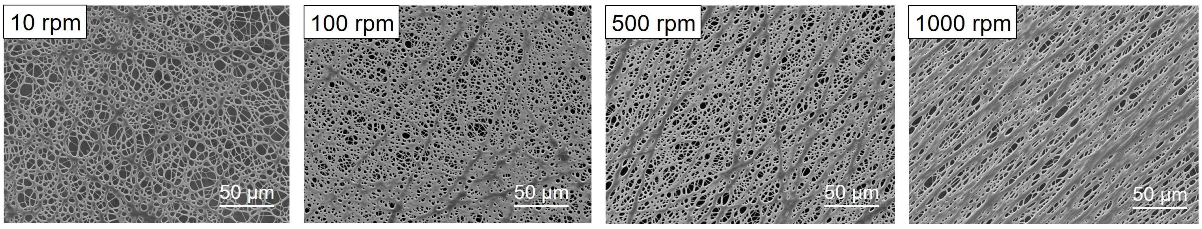


**Supplementary Fig. 10. SEM images of pLCE nanomeshes produced at different collector rotation speeds** (core flow rate = 0.3 mL $h^{-1}$; sheath flow rate = 0.4 mL $h^{-1}$). A rotation speed of 10 rpm was used in this study. Although a high rotation speed could induce nanofiber orientation, the fast nanofiber deposition led to incomplete UV crosslinking and a gradual loss of mesh porosity.

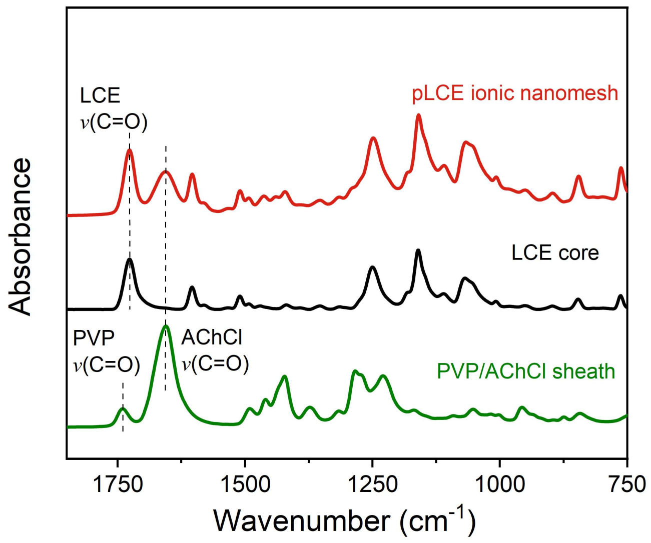


**Supplementary Fig. 11. ATR-FTIR spectra of LCE core, PVP/AChCl sheath and pLCE ionic nanomesh.** The spectrum of pLCE ionic nanomesh clearly shows characteristic peaks for *ν*(C=O) from both LCE and AChCl, confirming the successful integration of the sheath and core materials.

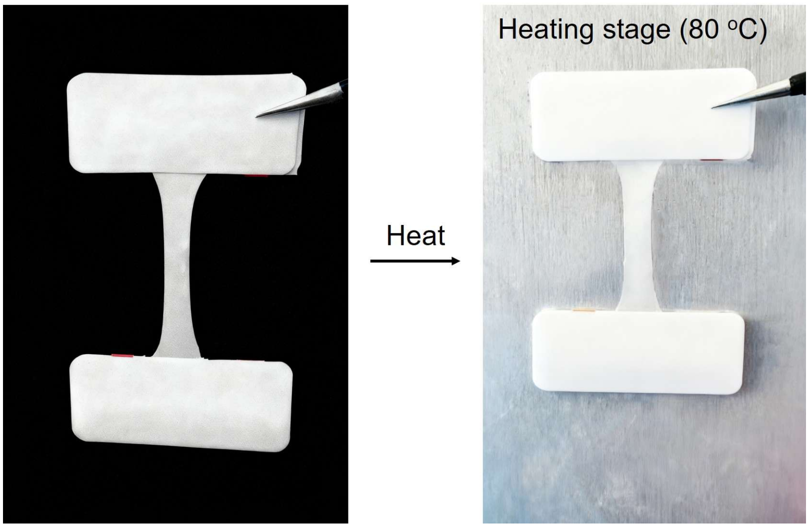


**Supplementary Fig. 12. Heat-induced contraction of stretched pLCE ionic nanomesh.** The ionic nanomesh rapidly contracted by ~17% when heated to 80 °C. This behavior results from the heat-induced liquid crystal-to-isotropic transition of the LCE, generating a contractive force to restore its original length.

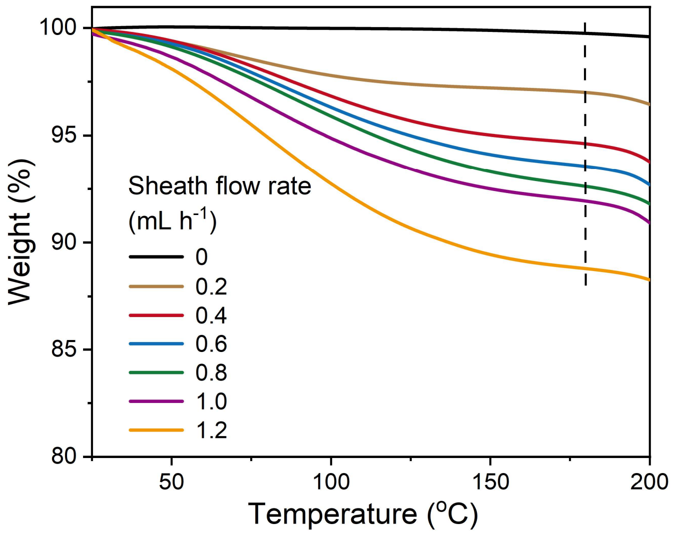


**Supplementary Fig. 13. TGA curves of pLCE ionic nanomeshes fabricated with varying sheath flow rates.** The water contents of ionic nanomeshes were determined by measuring the weight loss at 180 °C.

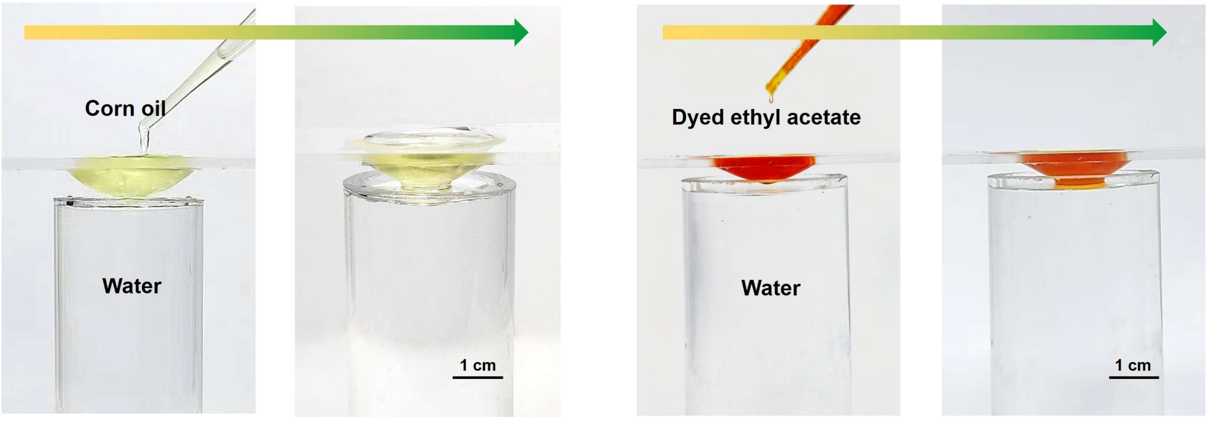


**Supplementary Fig. 14. Oil permeability of pLCE ionic nanomesh.** Hydrophobic liquids like corn oil and ethyl acetate (dyed with Sudan I) could not readily permeate the ionic nanomesh due to its hydrophilic surface.

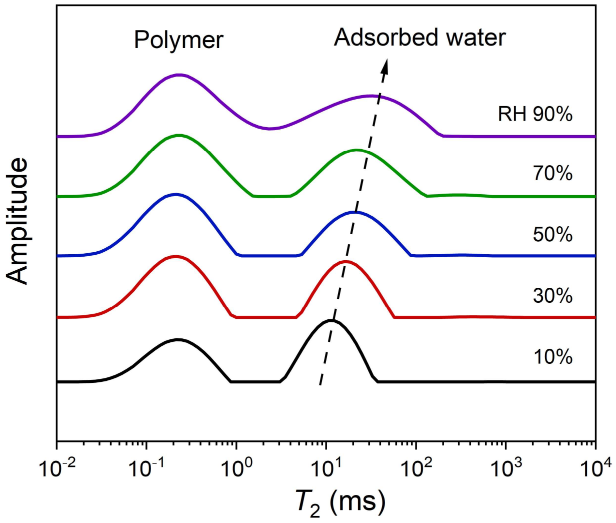


**Supplementary Fig. 15. Solid-state low-field $^1$H NMR spectra of pLCE ionic nanomesh at different humidities.** As humidity increased, the water peak broadened and shifted to a higher $T_2$, indicating an increasing proportion of mobile water transport.

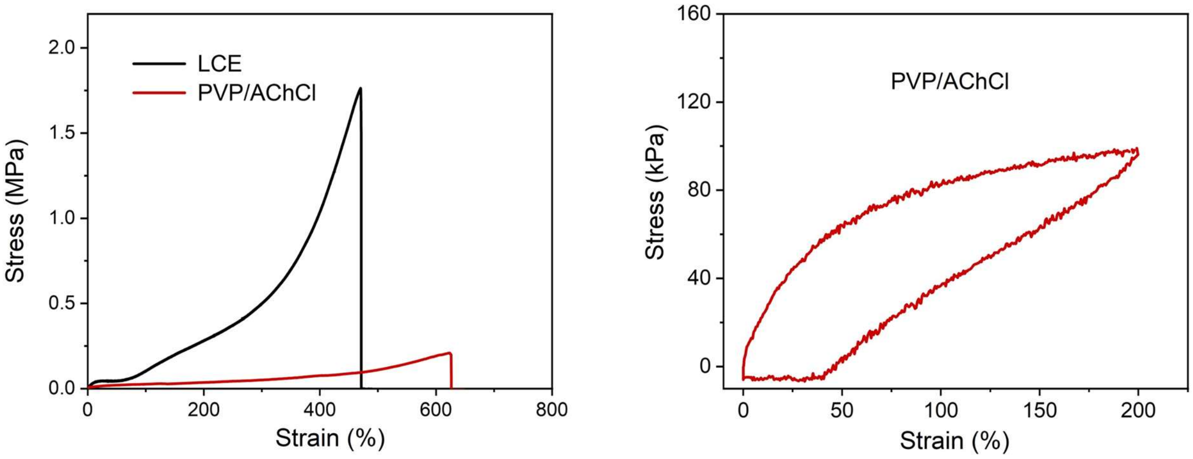


**Supplementary Fig. 16. Tensile curves of LCE and PVP/AChCl films**. The cast LCE and PVP/AChCl films, sharing the same compositions as the core and sheath materials in the pLCE ionic nanomesh, show Young's moduli of 326 kPa and 44 kPa, respectively. The loading-unloading curve of PVP/AChCl film reveals a more elastic response of the ionic sheath in the nanomesh, with a hysteresis ratio of 51%.

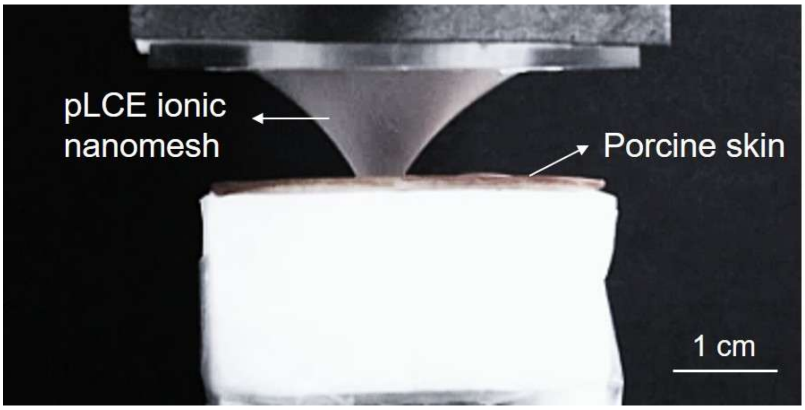


**Supplementary Fig. 17. Adhesion of pLCE ionic nanomesh to porcine skin.** Strong adhesion was observed when the ionic nanomesh was applied to adhere porcine skin.

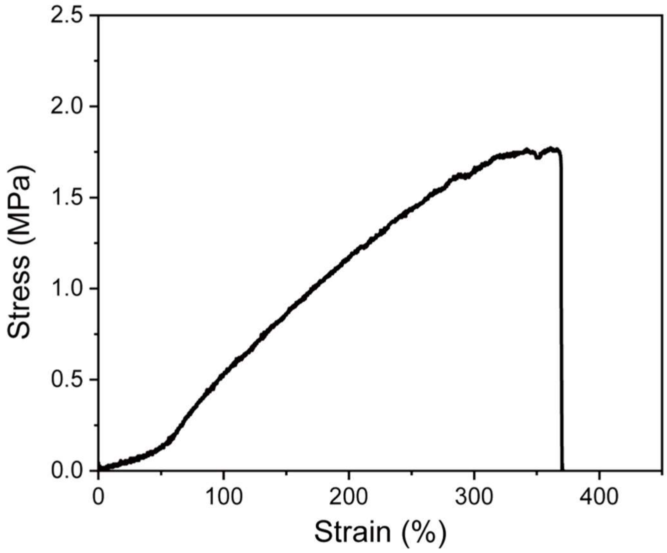


**Supplementary Fig. 18. Tensile curve of pLCE ionic nanomesh.** The pLCE ionic nanomesh exhibited a maximum elongation of 360%.

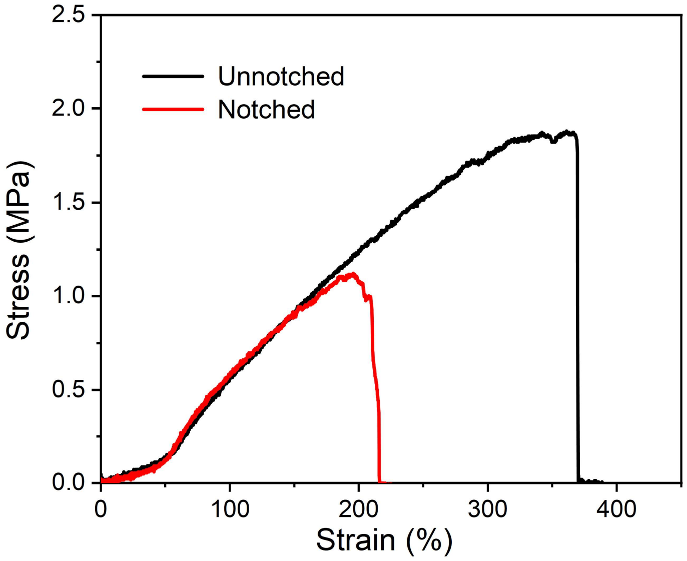


**Supplementary Fig. 19. Notch resistance of pLCE ionic nanomesh.** The ionic nanomesh with a 1/5 notch could still be stretched to over 200% strain, indicating its high notch resistance to tolerate mechanical damage.

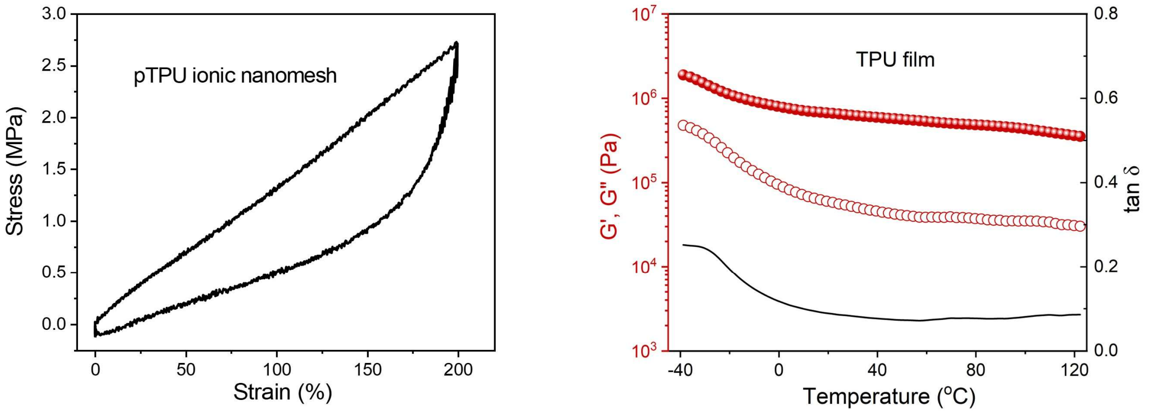


**Supplementary Fig. 20. Loading-unloading and temperature-sweep rheological curves of pTPU ionic nanomesh and TPU film.** The load-unloading tensile curve shows that pTPU ionic nanomesh possesses an elastic response with a low hysteresis ratio of 54%. This elastic behavior is consistent with the temperature-sweep rheological results, where the TPU film remained purely elastic across the measured temperature range, exhibiting small tan δ values (tan δ = 0.08 at 25 °C).

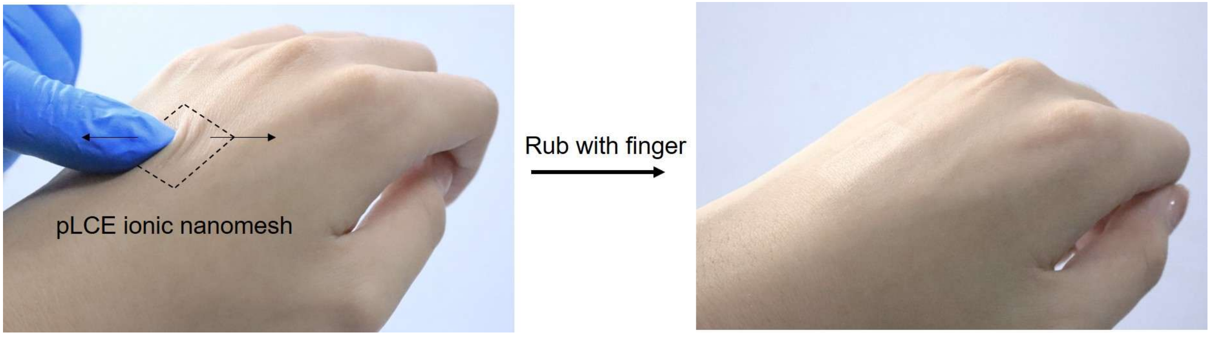


**Supplementary Fig. 21. Scratch resistance of pLCE ionic nanomesh adhered on hand skin.** The ionic nanomesh remained intact after repeated rubbing with a finger, demonstrating its good scratch resistance.

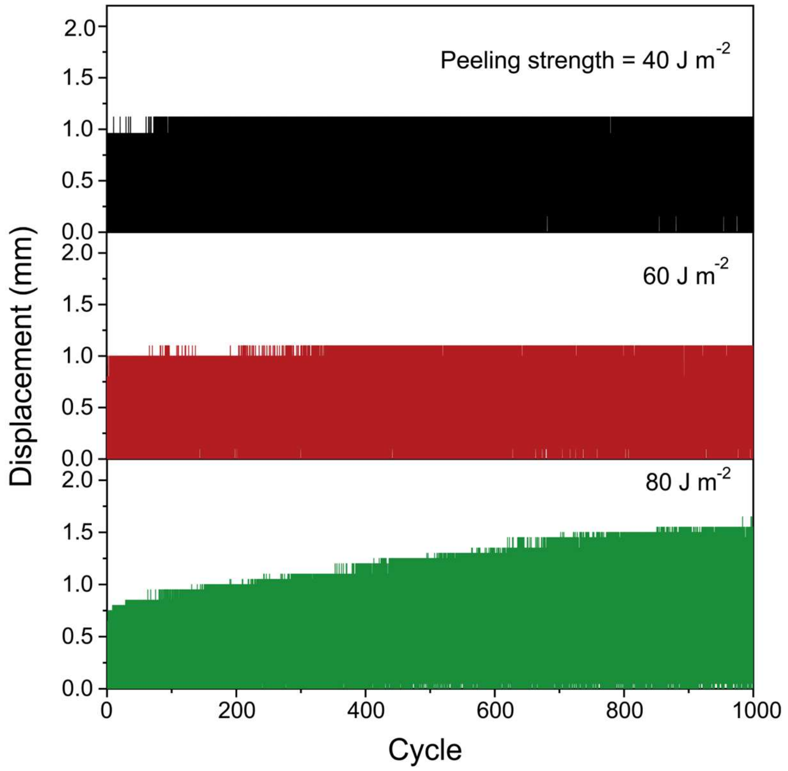


**Supplementary Fig. 22. Cyclic 90° peeling curves of pLCE ionic nanomesh at different peeling strengths.** Fatigue interfacial failure for pLCE ionic nanomesh was observed only when the peeling strength reached 80 J $m^{-2}$.

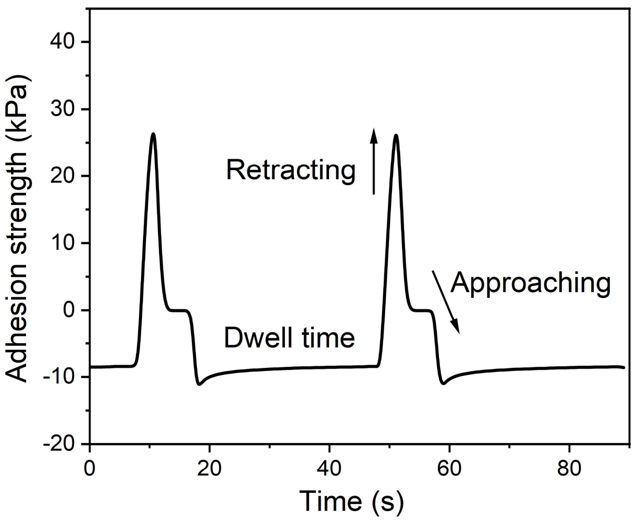


**Supplementary Fig. 23. Probe-tack curve of pLCE ionic nanomesh on porcine skin.** For this test, the pLCE ionic nanomesh was attached to the upper plate (as the probe), lowered onto the porcine skin at 20 mm min$^{-1}$, maintained in contact under a 0.5 N force for 30 s, and then separated at a lift.

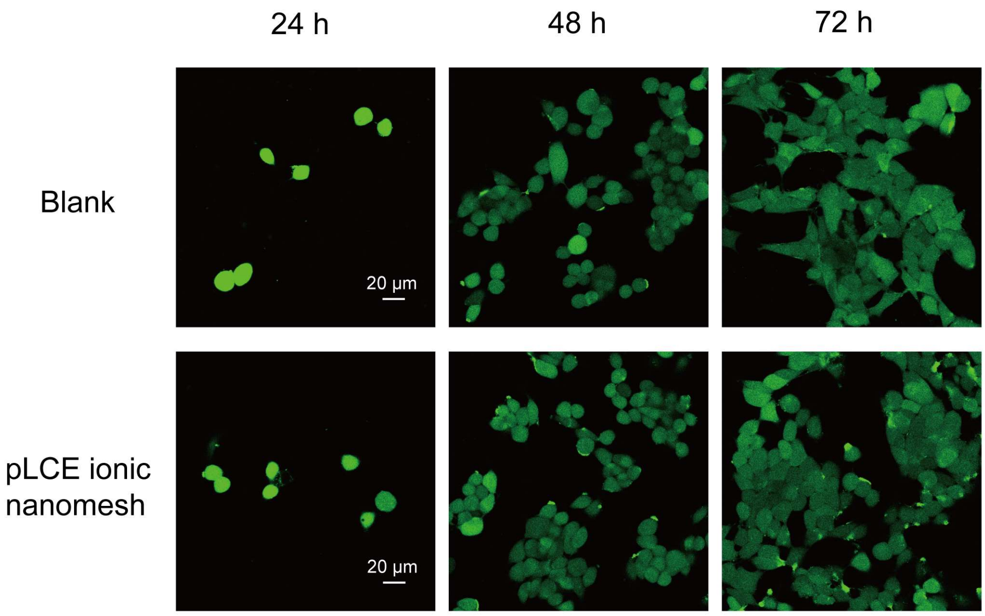


**Supplementary Fig. 24. CLSM images of cell proliferation in pLCE ionic nanomesh from dead/live staining experiments.** The results show the biocompatibility of the leaching solutions of pLCE ionic nanomesh (1 mg mL$^{-1}$) over a period of 24 to 72 h.

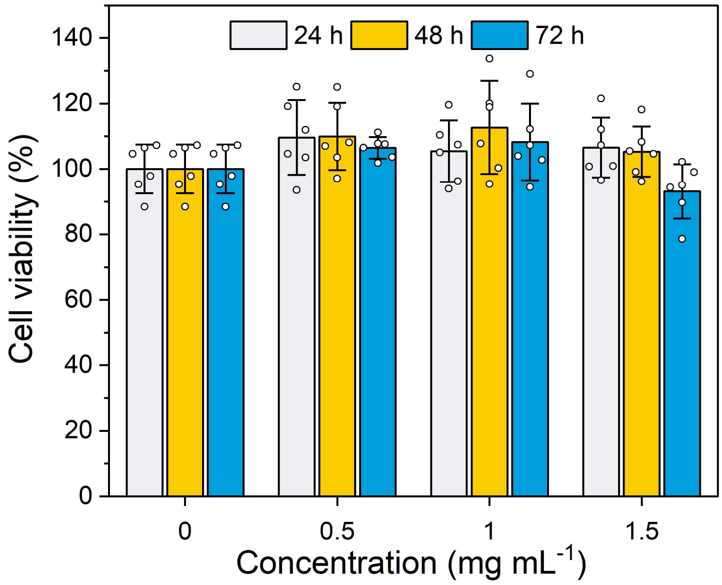


**Supplementary Fig. 25. L929 cytotoxicity results of pLCE ionic nanomesh.** Data are presented as the mean values ± SD, $n$ = 6 independent samples.

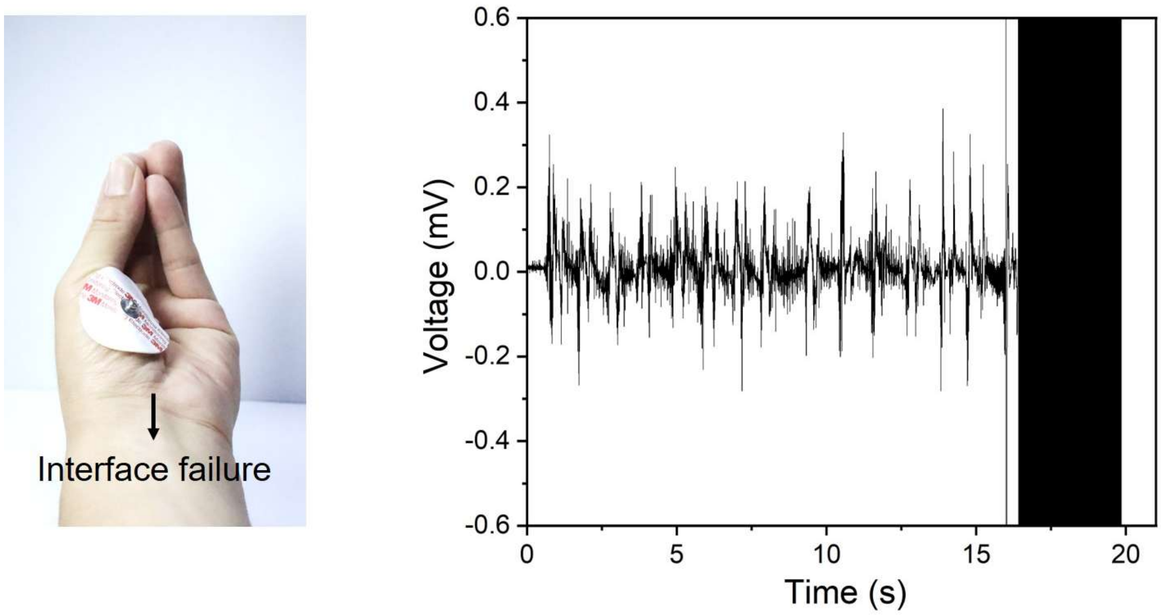


**Supplementary Fig. 26. Real-time EMG monitoring of hand gestures with commercial Ag/AgCl gel electrodes.** Commercial Ag/AgCl gel electrodes experienced interfacial failure, leading to motion artifact-induced signal degradation, typically within 20 cycles of making a claw gesture.

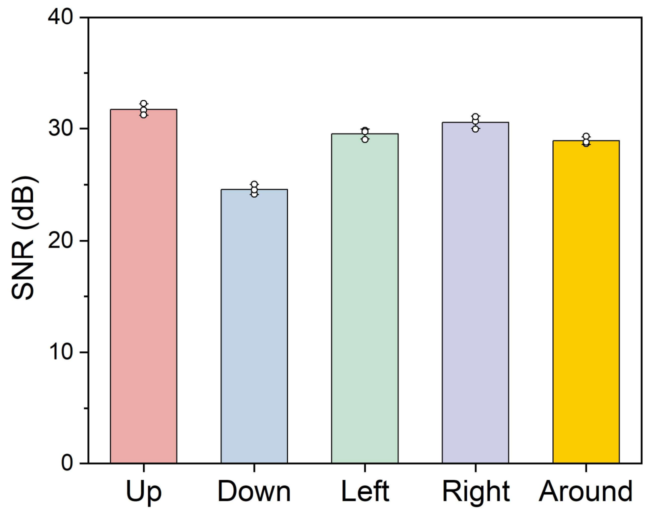


**Supplementary Fig. 27. SNRs of pLCE ionic nanomesh electrodes during various thumb movements.** Data are presented as the mean values ± SD, $n = 3$ independent samples.

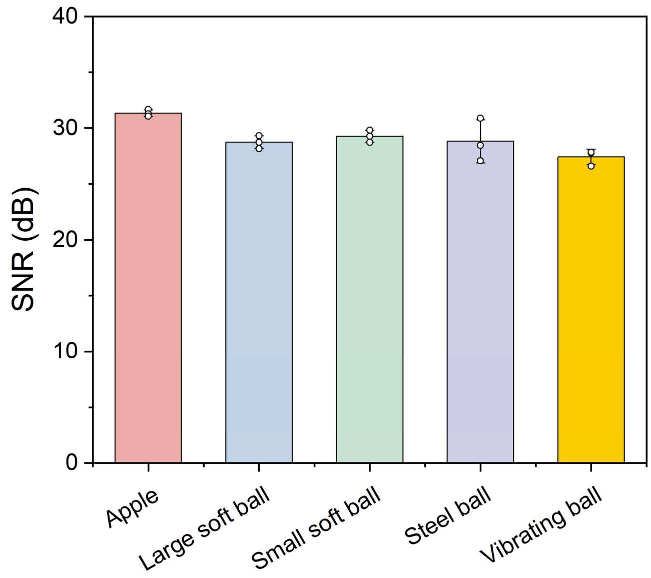


**Supplementary Fig. 28. SNRs of pLCE ionic nanomesh electrodes by repeated grasping of different objects.** Data are presented as the mean values ± SD, $n = 3$ independent samples.

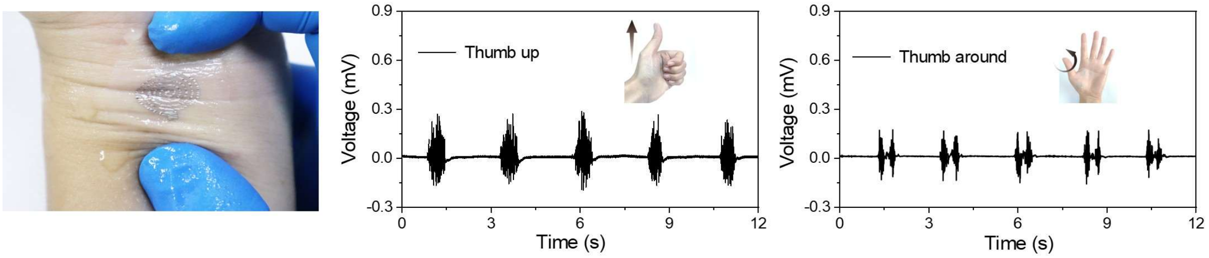


**Supplementary Fig. 29. Real-time EMG monitoring recorded with pLCE ionic nanomesh electrodes on sweaty skin.** The ionic nanomesh electrodes adhered firmly to sweaty skin, and consistently provide high-quality EMG signals.

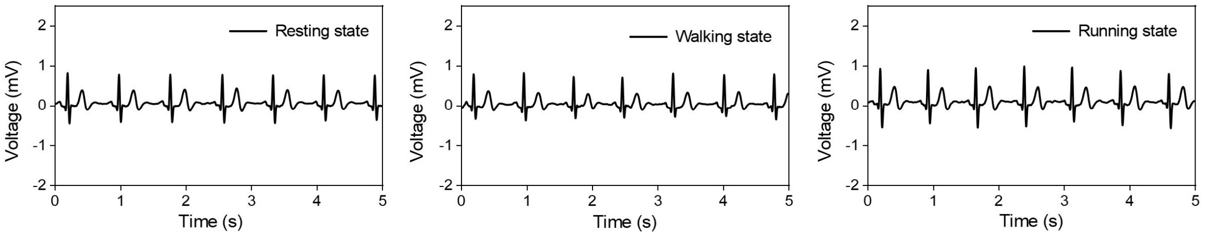


**Supplementary Fig. 30. Real-time ECG monitoring recorded with pLCE ionic nanomesh electrodes under resting, walking, and running conditions.** Highly reliable data were obtained under different conditions, demonstrating the reliability of our electrodes in collecting ECG signals.

**Supplementary Table 1. Comparison of moisture permeability among typical breathable on-skin materials.**

| Materials | Thickness (μm) | WVTR (g $m^{-2}$ $day^{-1}$) | Ref. |
|---|---|---|---|
| pLCE ionic nanomesh | 8 | 1245 | This work |
| MXene/PDMS/PDA/PU nanocomposite | ~200 | 1026 | [S2] |
| PAAm-alginate hydrogel | 50 | 1890 | [S3] |
| EGaIn-SBS mat | 320 | 724 | [S4] |
| PVA hydrogel nanomesh | 6 | ~2000 | [S5] |
| PU-gelatin hydrogel | 2.7 | 1252.3 | [S6] |
| liquid-metal-containing nanofibre membrane | 21 | 2941 | [S7] |
| 3D liquid diode | 650 | ~1700 | [S8] |
| Wet-adaptive electronic skin | ~300 | 852 | [S9] |
| IP6-PVA ion-conductive elastomer | 150 | 448.8 | [S10] |

**Supplementary References**


[S1] Yao, M., Wu, B., Feng, X., Sun, S. & Wu, P. A Highly Robust Ionotronic Fiber with Unprecedented Mechanomodulation of Ionic Conduction. *Adv. Mater.* **33**, 2103755 (2021).

[S2] Wang, X. et al. Biocompatible and breathable healthcare electronics with sensing performances and photothermal antibacterial effect for motion-detecting. *npj Flexible Electron.* **6**, 95 (2022).

[S3] Cheng, S. et al. Ultrathin hydrogel films toward breathable skin-integrated electronics. *Adv. Mater.* **35**, 2206793 (2023).

[S4] Ma, Z. et al. Permeable superelastic liquid-metal fibre mat enables biocompatible and monolithic stretchable electronics. *Nat. Mater.* **20**, 859-868 (2021).

[S5] Liu, L. et al. Sweat-activated conductive hydrogel nanomesh for breathable, long-term electrophysiological monitoring and human-centric interfaces. *Matter* **9**, 102428 (2025).

[S6] Wang, Y. et al. A 2.7-μm-thick robust, permeable, and antifreezing hydrogel electrode for long-term ambulatory health monitoring. *Sci. Adv.* **11**, eadt2286 (2025).

[S7] Zheng, S. et al. Pressure-stamped stretchable electronics using a nanofibre membrane containing semi-embedded liquid metal particles. *Nat. Electron.* **7**, 576–585 (2024).

[S8] Zhang, B. et al. A three-dimensional liquid diode for soft, integrated permeable electronics. *Nature* **628**, 84-92 (2024).

[S9] Chen, F. et al. Wet-Adaptive Electronic Skin. *Adv. Mater.* **35**, 2305630 (2023).

[S10] Niu, W., Tian, Q., Liu, Z. & Liu, X. Solvent-Free and Skin-Like Supramolecular Ion-Conductive Elastomers with Versatile Processability for Multifunctional Ionic Tattoos and On-Skin Bioelectronics. *Adv. Mater.* **35**, 2304157 (2023).
