# Supplementary material for: Self-compliant ionic nanomesh for gas-permeable and stress-free on-skin electronics
Source: Nat Commun. 2025 Nov 20;16:11510. doi: 10.1038/s41467-025-66512-2 (PMC12749131; doi:10.1038/s41467-025-66512-2)
Supplement: Supplementary file 2 — Description of Additional Supplementary Files [file 41467_2025_66512_MOESM2_ESM.pdf]

Description of Additional Supplementary Files

File name: Supplementary Movie 1

Description: Permeability of pLCE ionic nanomesh

File name: Supplementary Movie 2

Description: Breathable pLCE ionic nanomesh

File name: Supplementary Movie 3

Description: Fast water transport through pLCE ionic nanomesh

File name: Supplementary Movie 4

Description: Self-compliance of pLCE ionic nanomesh
